# Supplementary material for: Motivation of nursing undergraduate program entrants: A scoping review protocol
Source: PLoS One. 2025 Jan 3;20(1):e0311426. doi: 10.1371/journal.pone.0311426 (PMC11698347; doi:10.1371/journal.pone.0311426)
Supplement: S1 File — (DOC) [file pone.0311426.s001.doc]

**PRISMA-P (Preferred Reporting Items for Systematic review and Meta-Analysis Protocols) 2015 checklist: recommended items to address in a systematic review protocol***

| Section and topic | Item No | Checklist item |
| --- | --- | --- |
| ADMINISTRATIVE INFORMATION | | |
| Title: |  |  |
| Identification | 1a | Scoping review protocol |
| Update | 1b | Does not apply to this study |
| Registration | 2 | Open Science Framework (OSF) platform under DOI 10.17605/OSF.IO/EJNGY |
| Authors:  Contact: |  | Leonila Santos de Almeida Sasso^1^ ([leosaalm988@gmail.com](mailto:leosaalm988@gmail.com)); Ana Caroline dos Santos Costa^2^([a.carolinecosta@hotmail.com](mailto:a.carolinecosta@hotmail.com));  Ana Maria Rita Pedroso Vilela Torres de Carvalho Engel^2^([anavilelaengel@gmail.com](mailto:anavilelaengel@gmail.com)); Emília Batista Mourão Tiol^2^ ([emiliamouraotiol@gmail.com](mailto:emiliamouraotiol@gmail.com)); Fabrício Renato Teixeira Valença^1^([fabricio.valenca1@gmail.com](mailto:fabricio.valenca1@gmail.com)); Natalia Almeida de Arnaldo Silva Rodrigues Castro^1^([natalia.castro@edu.famerp.br](mailto:natalia.castro@edu.famerp.br)); João Daniel de Souza Menezes^1^([joao.menezes@edu.famerp.br](mailto:joao.menezes@edu.famerp.br)); Cíntia Canato Martins^3^([cintiacanato1@gmail.com](mailto:cintiacanato1@gmail.com)); Carlos Dario da Silva Costa^1^([carlos.costa@edu.famerp.br](mailto:carlos.costa@edu.famerp.br)); Maria Aurélia da Silveira Assoni^4^([aureliaassoni@yahoo.com.br](mailto:aureliaassoni@yahoo.com.br)); Wiliam Donegá Martinez^1^([william.martinez@edu.famerp.br](mailto:william.martinez@edu.famerp.br)); Patrícia da Silva Fucuta^5^([patricia@fukuta.com.br](mailto:patricia@fukuta.com.br)); Vânia Maria Sabadoto Brienze^1^(vania.brienze@hospitaldebase.com.br), Alba Regina de Abreu Lima^1^ ([alba.lima09@gmail.com](mailto:alba.lima09@gmail.com)), Júlio César André^1^(julio.andre@edu.famerp.br) . |
| Contact | 3a | 1. FAMERP- Faculty of Medicine of São José do Rio Preto, Brazil  2. UNIFUNEC - University Center of Santa Fé do Sul, Brazil  3. UNIP- Paulista University, Brazil  4. HA - Cancer Hospital of Barretos, Brazil  5. FACERES - Faceres Medical School, São José do Rio Preto, Brazil |
| Contributions | 3b | The guarantor of the review  Leonila Santos de Almida Sasso (Corresponding author; Conceptualization Writing – original draft);  Ana Caroline dos Santos Costa (Investigation);  Maria Rita Pedroso Vilela Torres de Carvalho Engel (Formal analysis);  Emília Batista Mourão Tiol (Supervision);  Fabrício Renato Teixeira Valença (Visualization);  Natalia Almeida de Arnaldo Silva Rodrigues Castro (Validation);  João Daniel de Souza Menezes (Resources);  Cíntia Canato Martins (Resources);  Carlos Dario da Silva Costa (Writing – review & editing);  Maria Aurélia da Silveira Assoni (Conceptualization);  Wiliam Donegá Martinez (Methodology);  Patrícia da Silva Fucuta (Data curation);  Vânia Maria Sabadoto Brienze (Supervision);  Alba Regina de Abreu Lima (Supervision);  Júlio César André (Project administration). |
| Amendments | 4 | Important protocol amendments will be made by the publishing platform. |
| Support: |  |  |
| Sources | 5a | The authors received no specific funding for this work |
| Sponsor | 5b | Review not financed and/or sponsor |
| Role of sponsor or funder | 5c | Does not apply to this study |
| INTRODUCTION | | |
| Rationale | 6 | Understanding the status quo of motivation among entrants to nursing programs can help identify students' academic profiles and the degree of resilience and perseverance needed to complete their studies and advance as professionals in the field. The type of motivation students have makes it possible to predict their levels of commitment, the risk of dropping out of the program and their academic progress and whether it falls below or exceeds expectations. Existing data on the motivation of nursing students makes it possible to identify any gaps in knowledge and enables an analysis of students' levels of commitment, the risk of dropping out and the influence of motivation on academic progress. |
| Objectives | 7 | To identify and showcase the available literature on the status quo of motivation among nursing program entrants, and to identify the key characteristics of this motivation that may predict their commitment level, risk of program dropout, and academic progression. |
| METHODS | | |
| Eligibility criteria | 8 | This review will consider all documents retrieved as Full-text articles; studies with quantitative and qualitative methodologies; primary research; original articles; theoretical explorations; experience reports; clinical study articles; case studies; normative, integrative and systematic reviews; meta-analyses; meta-syntheses; monographs; theses; and dissertations that contain relevant information on the motivation of nursing program entrants. |
| Information sources | 9 | Electronic databases: Cumulative Index to Nursing and Allied Health Literature (CINAHL); Latin American and Caribbean Health Sciences Literature (LILACS), Lilacs Esp, National Library of Medicine (PubMed), ScienceDirect, and on the Web of Science platform. |
| Search strategy | 10 | Ten MeSH descriptors were chosen and searched together: Students, Nursing, Pupil Nurses, Motivation, Disincentive, Expectation, Incentive, Education, Nursing, Nursing Education in all six bases. The following Boolean operators were used for combining the descriptors: AND, OR, and NOT. The search covered the period January 2017 to December 2023. |
| Study records: |  |  |
| Data management | 11a | The extraction will be carried out using Rayyan® extraction processes. The data will be recorded and organized into Excel sheets, considering bibliographic information; country of origin of the study; type of study; Population; location of the study; type of motivation identified in the study and Results found. |
| Selection process | 11b | To align the eligibility criteria among the researchers, the title and abstract of 25 random articles were analyzed by three of the researchers. There was a 100% agreement concerning the inclusion and exclusion of the articles. Disagreements regarding the inclusion or exclusion of the articles were discussed until a consensus was reached. |
| Data collection process | 11c | After the articles’ selection, the main researcher will create a form for data extraction, which will be filled in after the articles’ full analysis. |
| Data items | 12 | The extracted data will include specific details such as journal and year of publication, title, author, country, type of study, participants, concept, context, type of motivation. |
| Outcomes and prioritization | 13 | The main outcome sought will be the on the types of motivation observed among nursing program entrants and will include any key findings, trends, patterns, or notable gaps we have identified in the existing literature concerning social media and teaching and learning. |
| Risk of bias in individual studies | 14 | The results will be submitted to software and proper statistical analyses to avoid the risk of bias. The results will be evaluated by peers to identify probable missing bias. |
| Data synthesis | 15a | Study data will be quantitatively synthesised in descriptive form. |
|  | 15b | Our analysis will primarily focus on the types of motivation observed among nursing program entrants and will include any key findings, trends, patterns, or notable gaps we have identified in the existing literature concerning social media and teaching and learning. The methods of handling data and methods of combining data from studies will be chosen posteriorly, guided by the results founded, in the appropriate manner. |
|  | 15c | Does not apply to this study |
|  | 15d | Our reporting of the search strategy outcomes and other aspects will adhere to the PRISMA-ScR guidelines |
| Meta-bias(es) | 16 | Does not apply to this study |
| Confidence in cumulative evidence | 17 | Does not apply to this study |

*** It is strongly recommended that this checklist be read in conjunction with the PRISMA-P Explanation and Elaboration (cite when available) for important clarification on the items. Amendments to a review protocol should be tracked and dated. The copyright for PRISMA-P (including checklist) is held by the PRISMA-P Group and is distributed under a Creative Commons Attribution Licence 4.0.**

*From: Shamseer L, Moher D, Clarke M, Ghersi D, Liberati A, Petticrew M, Shekelle P, Stewart L, PRISMA-P Group. Preferred reporting items for systematic review and meta-analysis protocols (PRISMA-P) 2015: elaboration and explanation. BMJ. 2015 Jan 2;349(jan02 1):g7647.*
