# Supplementary material for: Motivation of nursing undergraduate program entrants: A scoping review protocol
Source: PLoS One. 2025 Jan 3;20(1):e0311426. doi: 10.1371/journal.pone.0311426 (PMC11698347; doi:10.1371/journal.pone.0311426)
Supplement: Attachment — Submitted filename: review file.docx [file pone.0311426.s002.docx]

dear Authors,

I congratulate you on this very expertly written manuscript. I would just like you to add the importance of the publication of this study protocol in the introduction or in the conclusion.

Kind regards,

Marija Spevan, Msn, PhD
