# Supplementary material for: Motivation of nursing undergraduate program entrants: A scoping review protocol
Source: PLoS One. 2025 Jan 3;20(1):e0311426. doi: 10.1371/journal.pone.0311426 (PMC11698347; doi:10.1371/journal.pone.0311426)
Supplement: Attachment — Submitted filename: Response to reviewers.docx [file pone.0311426.s003.docx]

Dear Esteemed Reviewers,

We extend our sincere gratitude for your invaluable contributions and the time dedicated to reviewing our manuscript. Your insightful observations and suggestions have been instrumental in enhancing the quality and clarity of our work. We present below our responses to each point raised, along with the corresponding modifications implemented in the manuscript.

**Reviewer 1:**

**1. The introduction is lengthy and could be shortened.**

Response: We appreciate your observation. We have significantly condensed the introduction, retaining only essential information. The revised section can be observed at the beginning of the Introduction:

"In today's health professions, professionals are expected to maintain positive attitudes toward patients. However, there is a noticeable decline in humanistic values among future health professionals, along with reduced idealism and empathy, increased skepticism, and diminished feelings of accomplishment and satisfaction with their career choices. [...]"

2. It is preferable to state the research purpose as a sentence rather than a question. Please correct the study purpose in both the abstract and at the end of the introduction. Response: We have implemented your suggestion and reformulated the study purpose as an affirmative statement. In the abstract, the modification can be seen in the following excerpt:

"The objective of this review is to identify and present the information available in the literature on the status quo of motivation among nursing program entrants."

In the introduction, we have added the following paragraph at the end:

"The objective of this study is to identify and showcase the available literature on the status quo of motivation among nursing program entrants, and to identify the key characteristics of this motivation that may predict their commitment level, risk of program dropout, and academic progression."

3. The necessity for conducting this research is not well explained in the introduction. Response: We appreciate you highlighting this gap. We have incorporated a paragraph in the introduction to better elucidate the need for this research:

"International research on motivation for learning among nursing students has intensified in the past decade. The primary focus of international research is on identifying the type of motivation that has the most impact on nursing students, including EM and IM, and the positive and negative associations between types of motivation and student grades and academic outcomes [22]. However, there is still a gap in understanding the specific motivations of nursing program entrants, which could provide valuable insights into student retention, academic performance, and future professional commitment."

4. Reference tables one, two, and three in the text. Response: We appreciate your observation. We have included references to the tables in the text, as can be seen in the following excerpts:

"The search strategy is presented in Table 1." "Table 2 lists the databases to be used in the search and your search strategy." "The texts chosen by the two reviewers will have specific data collected through a data extraction process using a tool specifically designed for this purpose (see Table 3)."

5. The search strategy overlaps with the content of Table One. Please remove one. Response: We appreciate your suggestion. We have removed the detailed description of the search strategy from the main text and retained only the reference to Table 1, which now contains all necessary information about the search strategy.

**Reviewer 2:**

**1. I would like you to add the importance of publishing this study protocol in the introduction or conclusion.**

Response: We appreciate your suggestion. We have added a paragraph in the Justification section to address the importance of publishing this study protocol:

"Publishing this study protocol is crucial as it provides transparency in the research process, allows for peer review of the methodology before the study is conducted, and helps prevent duplication of efforts in the scientific community. It also serves as a reference point for future researchers interested in the motivation of nursing program entrants and contributes to the overall body of knowledge in nursing education."

Once again, we express our profound gratitude for your contributions, which have been essential in improving the quality of our manuscript. We trust that the implemented changes meet your expectations, and we remain at your disposal for any additional clarifications.

Yours sincerely, The Authors
