# Supplementary material for: Motivation of nursing undergraduate program entrants: A scoping review protocol
Source: PLoS One. 2025 Jan 3;20(1):e0311426. doi: 10.1371/journal.pone.0311426 (PMC11698347; doi:10.1371/journal.pone.0311426)
Supplement: Attachment — Submitted filename: review file2.docx [file pone.0311426.s004.docx]

Dear authors,

wave on accepting comments and suggestions. Congratulations on an excellent manuscript.

Kind regard,

Marija Spevan, MsN, PhD Biomed.
